# Updates on the research and development of absorbable metals for biomedical applications

**DOI:** 10.1007/s40204-018-0091-4

**Published:** 2018-05-22

**Authors:** Hendra Hermawan

**Affiliations:** Department of Mining, Metallurgical and Materials Engineering and CHU de Québec Research Center, Laval University, Quebec City, G1V 0A6 Canada

**Keywords:** Absorbable, Biodegradable, Corrosion, Iron, Magnesium, Metal, Zinc

## Abstract

**Abstract:**

Absorbable metals, metals that corrode in physiological environment, constitute a new class of biomaterials intended for temporary medical implant applications. The introduction of these metals has shifted the established paradigm of metal implants from preventing corrosion to its direct application. Interest toward absorbable metals has been growing in the past decade. This is proved by the rapid increase in scientific publication, progressive development of standards, and launching the first commercial products. Iron, magnesium, zinc, and their alloys are the current three absorbable metals families. Magnesium-based metals are the most progressing family with a large data set obtained from both basic and translational research. Iron-based metals are still facing a major challenge of low in vivo corrosion rate despite the significant efforts that have been put to overcome its weakness. Zinc-based metals are the new alternative absorbable metals with moderate corrosion rates that fall between those of iron and magnesium. This manuscript provides a brief review on the latest progress in the research and development of absorbable metals, the most important findings, the remaining challenges, and the perspective on the future direction.

**Graphical abstract:**

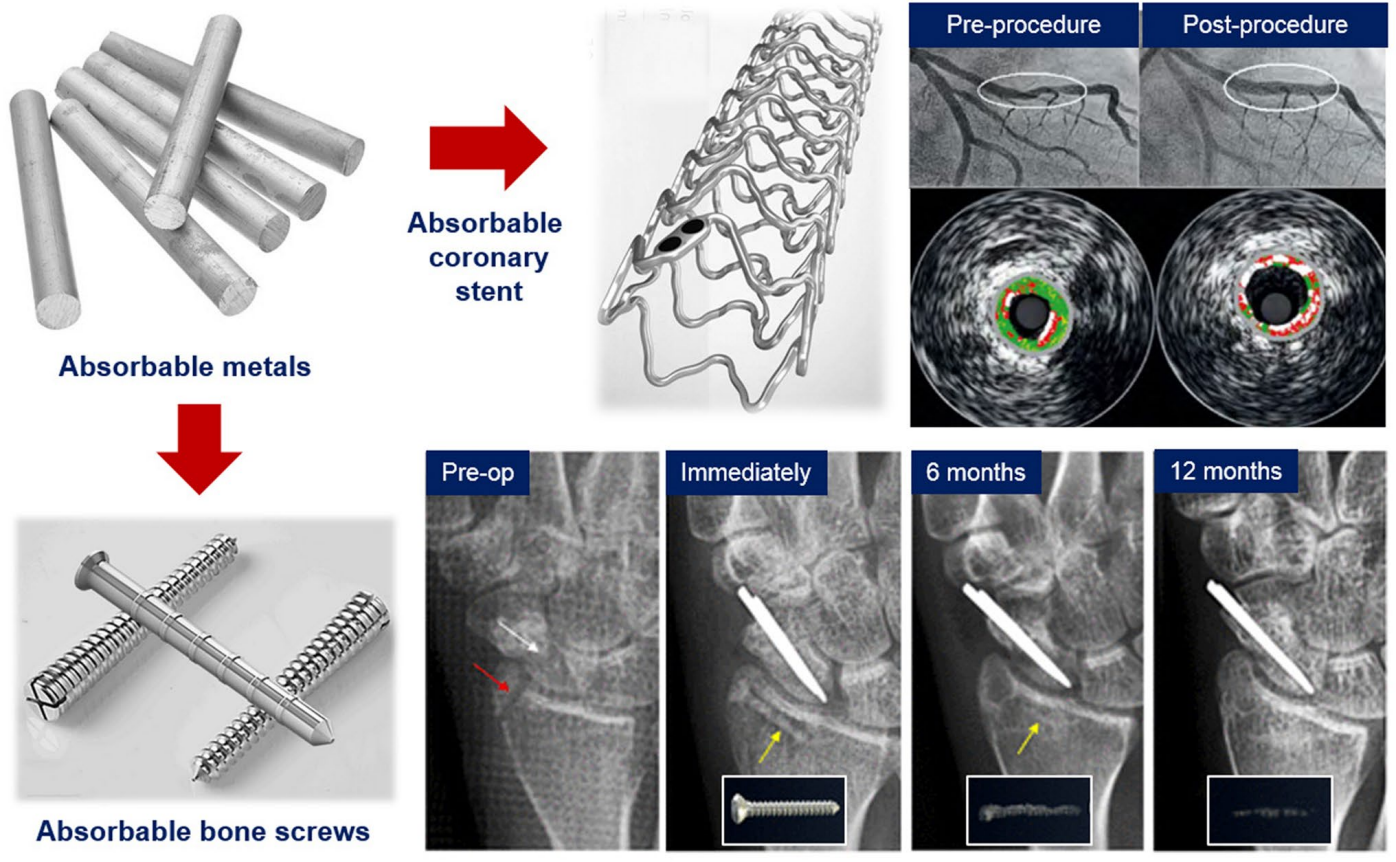

## Introduction

In recent years, there have been many media highlights on the emerging new medical technology based on the use of absorbable metals. One of them appeared as 2016 BBC Horizons report on “special” metal bone screw implanted in the broken finger bone of a male Korean patient (Horizons 2016). The patient returned to the hospital 4 months after the surgery with a smile on his face, because his broken bone had been healed and he did not need to go for a second surgery as the screw had disappeared. This innovative metal implant completely dissolves in the body after providing the needed function, thus eliminating the harmful potential effects of permanent implants. After decades of acknowledging that metal implants must be corrosion resistant, nowadays, corrosion is seen as an advantage. The interest toward these corrodible or absorbable metals has been rapidly growing. It is marked by the high increase of related scientific publications, the development of new ASTM and ISO standards, and the commercialization of three absorbable metal products.

Following the nomenclature by the ASTM F3160-16 standard (ASTM 2016a) and the suggestion by Liu et al. (2017b), the term “absorbable” is used in this article instead of the mostly known “biodegradable”. The prefix “bio” is not used, since it is redundant in the context of implant applications. This standard defines the term absorbable as “an initially distinct foreign material or substance that either directly or through intended degradation can pass through or be metabolized or assimilated by cells and/or tissue”. The term biodegradable is not a good fit for implantable devices. It causes confusion for the general audience, since it is broadly applied to composting and other natural processes that cause the breakdown of materials into chemical and/or particulate matter. In addition, in this article, the term “corrosion” is preferentially used over “degradation”, since it precisely indicates the electrochemical mechanism of metal dissolution that starts once a metal implant is exposed to the human/animal body fluid (in vivo) (Zheng et al. 2014; Agrawal et al. 2016).

Absorbable metals are expected to corrode gradually in vivo by generating an appropriate host response and then dissolve completely upon assisting tissue healing (Zheng et al. 2014). The absorbable metal family includes iron, magnesium, zinc, and their alloys. In a recent publication, iron-based stents were reported to demonstrate a good long-term biocompatibility when tested in animals (Lin et al. 2017). Stents made of magnesium alloys were clinically tested in human, and they showed a continuous desirable safety profile for 24 months, where no thrombosis or cardiac death was detected (Haude et al. 2017). Pure zinc stents were found to show a long-term steady corrosion process and biocompatibility in the vascular environments of rabbits (Yang et al. 2017). Aside from these three examples of most recent publications, there have been many more research articles related to absorbable metals published in the past 5 years. The latest comprehensive review on this subject was made by Zheng et al. (2014) in 2014, and some more partial reviews were published in the following years which focused on each metal and its application, i.e., magnesium, iron, or zinc for either cardiovascular or orthopaedic (Francis et al. 2015; Li et al. 2016; He et al. 2016; Zhao et al. 2017b; Mostaed et al. 2018). Therefore, this article aims to provide a new brief review on the latest progress of absorbable metals, to extract the most important findings, and to indicate the direction for future works. The work is presented in three main sections covering basic research, translational research, and development of standards with corrosion being the subject of interest that aligns the whole review. The author’s comments are added in the end of each section and are summarized in the perspective.

## Basic research

**Fig. 1:**
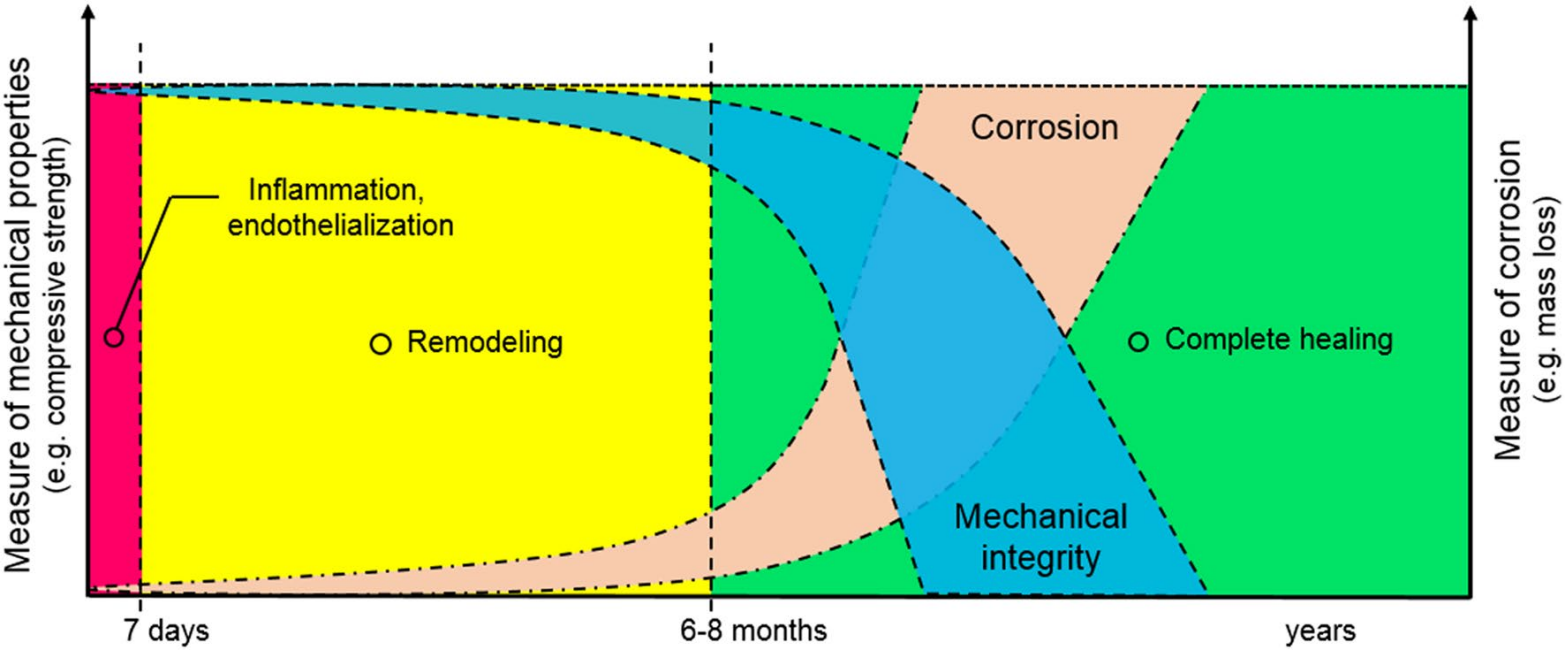
Illustration of the ideal compromise between mechanics and corrosion of absorbable metals for coronary stent application. Corrosion rate stays low during the first 6–8 months, while mechanical integrity stays high to allow vessel remodelling. Similar illustration is valid for absorbable bone implant, but the mechanical integrity should remain high for the first 3–6 months to allow bone repair process that takes place. Adapted with permission from Elsevier (Zheng et al. 2014)

In absorbable metal research, there is a constant search for biocompatible metals and alloys which show the optimum compromise between the level of mechanical and corrosion properties in the in vivo environment. Ideally, an implant made of these metals maintains its mechanical integrity during the necessary healing period while progressively corrodes (Fig. 1). The basic research in absorbable metals revolves around three main areas: (1) studying the toxicity of the metals both in vitro and in vivo as an indication of biocompatibility; (2) enhancing the mechanical properties of the metals through alloy design and metallurgical processes; (3) controlling the corrosion behavior of the metals by modifying its substrate or surface via coating and other surface treatments. The last two areas, mechanics and corrosion, often come together as they are the product of metallurgical processes. Controlling corrosion also helps to control the toxicity of the metals by achieving a balance between the release rate of corrosion products, i.e., metal ions, and the ability of the body to absorb and excrete them.

### The three families of absorbable metals

So far, absorbable metals are basically made of iron, magnesium, or zinc as the main ingredient (base metal). They are essential elements needed for proper metabolic function of human body, and are relatively compatible with human cells and tissues as confirmed in many in vitro and in vivo studies (Drynda et al. 2015; Myrissa et al. 2016; Kubásek et al. 2016; Marco et al. 2017; Wang et al. 2017a; Drelich et al. 2017). To improve both mechanical properties and corrosion behavior, the base metals are mostly alloyed with elements which are considered “non-toxic”. The first base metal, i.e., iron, has attractive mechanical properties approaching to those of the 316L stainless steels which is considered as a benchmark for metallic biomaterials. Indeed, pure iron has superior mechanical properties compared to pure magnesium and zinc (Table 1), making it a suitable candidate for implants which require a high structural strength such as coronary stents (Francis et al. 2015; He et al. 2016; Lin et al. 2016, 2017). Alloying with manganese turns the ferromagnetic iron into non-magnetic as a single austenitic phase is formed (Feng et al. 2016; Hufenbach et al. 2017). Iron was made into composites with calcium phosphate or calcium silicate that led to enhanced bioactivity in both in vitro and in vivo experiments (Ulum et al. 2015; Dehestani et al. 2016; Wang et al. 2017b).

**Table 1:** Representative and non-exhaustive examples of common absorbable metals and their properties

Metals	Yield strength (MPa)	Tensile strength (MPa)	Maximum elongation (%)	Corrosion rate* (mm/year)
Iron-based: Young’s modulus ~ 200 GPa, density ~ 7.8 g/cm^3^				
Pure iron as annealed	150	200	40	0.1
Fe-21Mn-0.7C as recrystallized	345	980	62	0.13
Fe-21Mn-0.7C-1Pd as recrystallized	360	970	64	0.21
Magnesium-based: Young’s modulus ~ 45GPa, density ~ 1.7 g/cm^3^				
Pure magnesium as extruded	30	100	7	8
Mg-1Ca as extruded	135	240	10	12.5
Mg-4Y-3RE (WE43) as extruded	180	280	10	4.3
Zinc-based: Young’s modulus ~ 100 GPa, density ~ 7.1 g/cm^3^				
Pure zinc as extruded	60	90	8	0.16
Zn-1Mg as extruded	170	250	10	0.12
Zn-3Cu-1Fe as extruded	210	270	20	0.13

Data were compiled from (Francis et al. 2015; Gong et al. 2015; Li et al. 2016; Agrawal et al. 2016; Zhao et al. 2017b; Yue et al. 2017; Mostaed et al. 2018)

*Corrosion rate data were collected from those having the most similar experiments, i.e., in simulated body fluid at 37 °C using polarization test, but they may not be directly comparable due to possible variation in specific testing condition and parameters

**Fig. 2:**
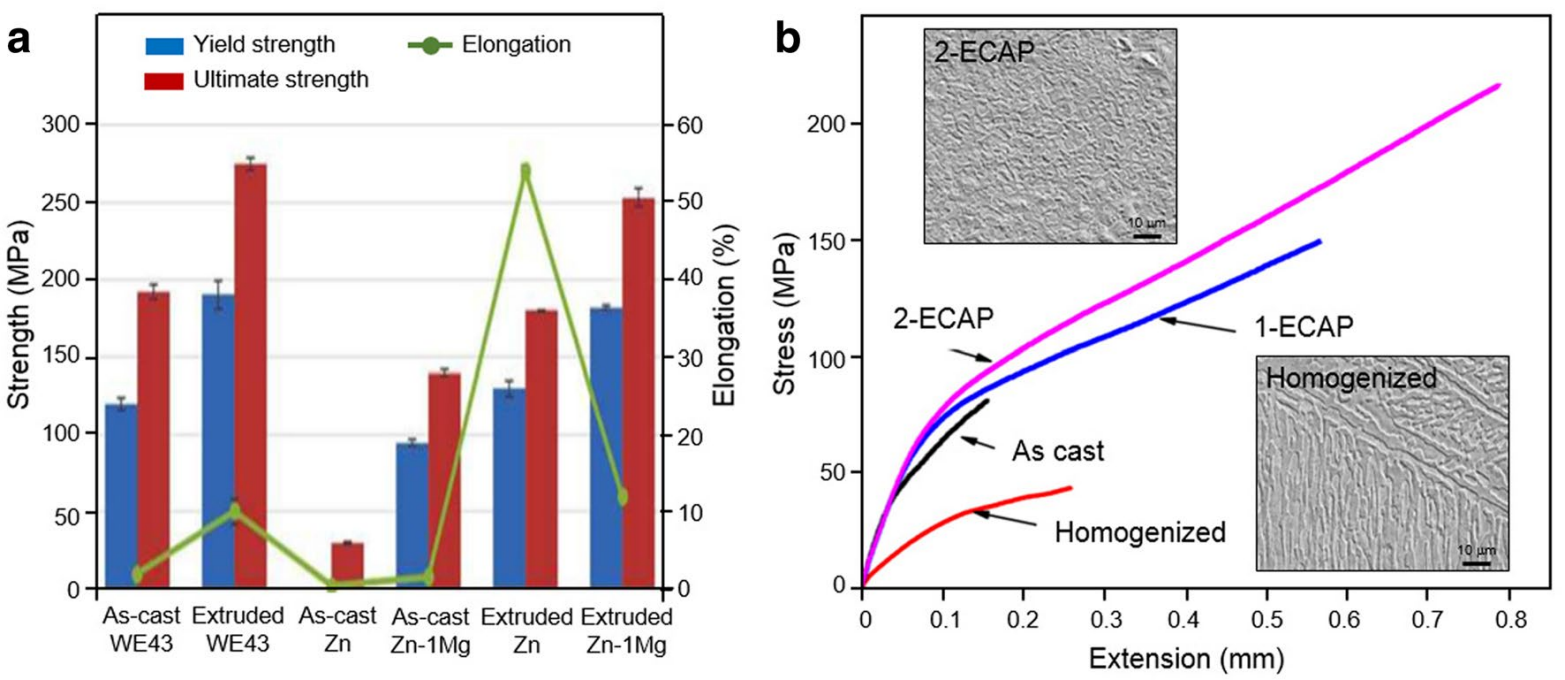
Enhancement of mechanical properties of magnesium and zinc alloys by: **a** extrusion of Zn−1 Mg at 200 °C and WE43 (Mg-RE) at 400 °C, both with extrusion ratio of 16:1 and 20-mm/s pressing rate; **b** ECAP of Zn−3 Mg alloy using 120° intersection angle and 200 °C heated dies at 1 mm/s pressing rate. Adapted with permission from John Wiley and Sons, and Elsevier (Gong et al. 2015; Dambatta et al. 2017)

The second base metal, i.e., magnesium, is a lightweight metal with density of 1.74 g/cm^3^, and it has a low elastic modulus of 40–45 GPa which is near to that of the bone (Li et al. 2016, Zhao et al. 2017b). Therefore, it is a very attractive candidate material for bone implants (Chaya et al. 2015; Han et al. 2015; Yu et al. 2017). Advanced alloying and processing techniques, such as thermomechanical treatment and severe plastic deformation, have improved the properties of magnesium-based metals (Sunil et al. 2016; Griebel et al. 2017) (Table 1). So far, the most complete literature coverage has been on magnesium-based metals. This has facilitated their clinical translation (Agrawal et al. 2016; Zhao et al. 2017b). The third base metal, i.e., zinc, has been lately added as a new family of absorbable metals (Liu et al. 2016b; Wang et al. 2016a; Levy et al. 2017). The cytocompatibility of zinc-based metals was studied in view of its applications for bone and vascular implants (Murni et al. 2015; Shearier et al. 2016; Guillory et al. 2016). Alloying zinc with magnesium and other trace elements, such as manganese, strontium, etc., improved the mechanical properties to those levels of some magnesium alloys (Gong et al. 2015; Mostaed et al. 2016; Liu et al. 2016a, b) (Table 1). Thermomechanical treatment, such as extrusion (Fig. 2a), was employed to improve both the strength and ductility of many magnesium and zinc alloys (Wang et al. 2014; Gong et al. 2015). The two alloys were also subjected to severe plastic deformation processing such as the equal channel angular pressing (ECAP) (Fig. 2b) to further enhance their mechanical properties (Mostaed et al. 2014; Dambatta et al. 2017).

### Attempts to improve the corrosion behavior

In terms of corrosion behavior, it is generally acknowledged that iron-based metals corrode slowly, magnesium-based metals corrode rapidly, and zinc-based metals corrode moderately (Li et al. 2016; He et al. 2016; Zhao et al. 2017b; Mostaed et al. 2018). Their corrosion rates in simulated body fluid indicate this trend (Table 1). A direct comparison done by Vojtech et al. (2015) showed corrosion rates of pure iron, pure zinc, and pure magnesium after 168 h of immersion in saline solution at 37 °C were 0.2, 0.6, and 4 mm/year, respectively. Many attempts have been done to control the corrosion behavior of absorbable metals, i.e., increasing corrosion rates of iron-based metals where its low rates become more evident under in vivo condition (Kraus et al. 2014; Drynda et al. 2015; Lin et al. 2017) and lowering those of magnesium-based metals as it also becomes problematic under in vivo condition (Shi et al. 2017; Cui et al. 2017; Liu et al. 2017a). Various advanced metallurgical processes have been used to achieve it. Basically, it was done by optimizing the composition and microstructure of the metals, either in the bulk, e.g. precise alloying, purification and improved manufacturing process, or on the surface, e.g. surface treatment process and protective coating (Table 2). Attempts to accelerate the corrosion rate of iron-based metals has been done by various methods such as alloying with manganese, palladium, silver, gallium, sulfur, and intermetallics (Čapek et al. 2016; Wang et al. 2017a; Hufenbach et al. 2017; Sotoudehbagha et al. 2018; Sikora-Jasinska et al. 2018), vacuum plasma nitriding process (Lin et al. 2016), implanting silver using a vapour vacuum arc technique (Huang et al. 2016), and making composite with polymers (Yusop et al. 2015; Cysewska et al. 2017; Qi et al. 2018). Slowing down the corrosion rate of magnesium-based metals has been addressed by various means such as purification from detrimental impurities (Hofstetter et al. 2015; Qu et al. 2017), alloying with calcium, zinc, rare earth elements (RE) and other elements (Zander and Zumdick 2015; Mao et al. 2017; Li et al. 2018), and coating with bioceramics or biopolymers (Hiromoto et al. 2015; Witecka et al. 2016; Su et al. 2016; Patil et al. 2017).

**Table 2:** Representative and non-exhaustive examples of method to improve the corrosion behavior of iron and magnesium

Materials and process	Results and rationale
Wang et al. (2017a) alloyed iron with gallium to form Fe_81_Ga_19_, (Fe_81_Ga_19_)_98_B_2_ and (Fe_81_Ga_19_)_99.5_ (TaC)_0.5_ and electrochemically tested their corrosion rate in simulated body fluid (SBF)	The addition of the more reactive gallium decreased corrosion resistance, but severe pitting corrosion was observed with the ternary B or TaC additives due to the formation of multi-phases on the surface of the alloys
Sikora-Jasinska et al. (2018) developed composite of iron and Fe/Mg_2_Si using a sequence process of mechanical milling, sintering, and multi-step hot rolling and conducted long-term immersion study in modified Hanks’ solution up to 100 days	The Mg_2_Si influenced the composition and decreased the stability of the formed oxide, hydroxide, carbonate, and phosphate films on the corroded surfaces, and thus, twofold increase of corrosion rate was measured for the composite compared to pure iron
Huang et al. (2016) implanted silver on pure iron using vapour vacuum arc technique and tested for corrosion electrochemically in Hanks’ solution	The microgalvanic action between Ag_2_O particles and iron matrix increased the corrosion rate (about two times faster)
Yusop et al. (2015) fabricated a composite of pure iron foam and poly(lactic-co-glycolic acid) (PLGA) via a vacuum infiltration technique	Corrosion rate in PBS increased approximately by two times in due to the dissolution effect of the polymer and its interfacial interaction with the iron substrate
Hofstetter et al. (2015) compared the corrosion behavior of ultrahigh purity magnesium made via casting and extrusion in SBF	The as cast exhibited significantly higher corrosion rates than the extruded due to the influence of iron-containing precipitates formed during casting
Zander et al. (2015) prepared ternary Mg–Zn–Ca alloys by casting without homogenisation and studied their corrosion behavior in Hanks’ solution	At Zn:Ca atomic ratio of 1.84, a zinc-enrichment of α-magnesium, decreased the electrical potential differences between the phases in Mg–Ca–Zn alloy and thus reduced the corrosion rate
Hiromoto et al. (2015) coated AZ31 alloy with hydroxyapatite (HA) and with octacalcium phosphate (OCP) and tested its corrosion by immersion in cell medium	The HA-coated AZ31 alloy corroded ~ 20% slower than the OCP-coated alloy. The OCP coating had higher resistance and stability than the HA coating
Witecka et al. (2016) studied the corrosion of ZM21 coated with four different polymers: PLGA, poly(l-lactide acid) (PLLA), poly(3-hydroxybutyrate) (PHB), poly(3-hydroxybutyrate-*co*-3-hydroxyvalerate) (PHH)	After 4 weeks of immersion in cell medium, the corrosion rate of ZM21 decreased more when coated with PLLA, PHB and PHH compared to that with PLGA due to the lower water permeability of the three former coatings

In addition, using porous structure of iron was recently studied as a way to control its corrosion rate while targeting a new potential application for bone scaffolds (Heiden et al. 2016; Feng et al. 2017). This actually opened a new direction of using porous absorbable metals as alternative to polymers for scaffolds by exploiting mostly their superior mechanical properties over biodegradable polymers. The emerging additive manufacturing technology helps to advance the design and process of ideal topological porous metals suited for bone scaffolds and orthopaedic implants (Wang et al. 2016c; Gordeladze et al. 2017). The high strength and slow corrosion of iron give a higher degree of freedom to vary its surface area/weight ratio for controlling the corrosion rate and matching the different strength and flexibility requirement for bone scaffolds (Yusop et al. 2015; Alavi et al. 2017; Yang et al. 2018). Differently, the high surface area of porous structure increases the challenge of controlling the rapid corrosion of magnesium scaffolds (Aghion and Perez 2014; Cheng et al. 2016). As for its solid form, alloying, composite fabrication, and surface coatings are the methods used to control the corrosion of porous magnesium (Yazdimamaghani et al. 2017). As for zinc-based metals, Zhao et al. (2016d) suggested porous zinc for low load-bearing bone scaffolds, since they observed its good corrosion resistance in simulated body fluid and its ability to induce CaP precipitation during immersion tests. The following section describes in vivo corrosion behavior of absorbable metals during implantation in animals.

### In vivo corrosion behavior

**Fig. 3:**
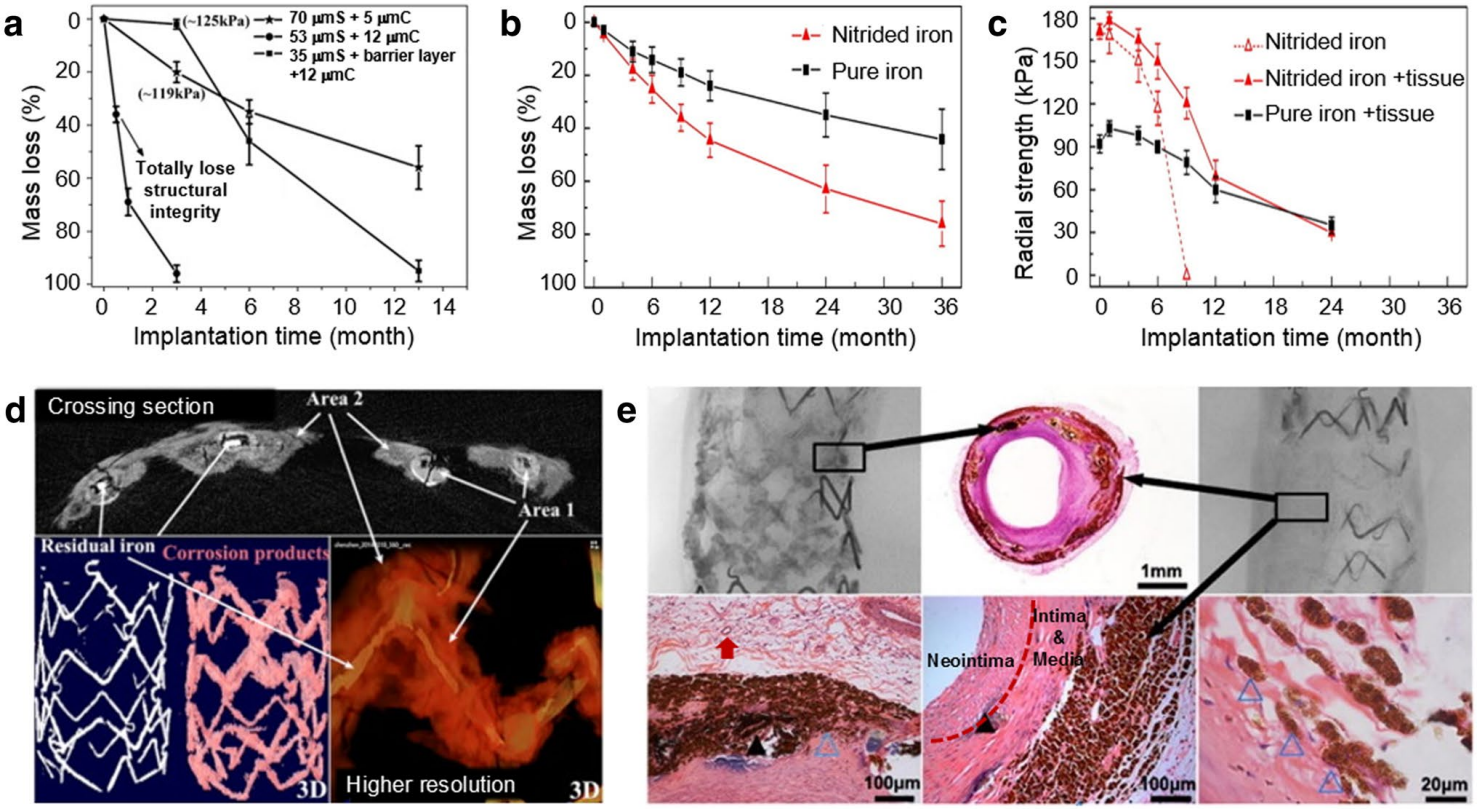
In vivo corrosion profile of various iron stents: **a** mass loss of coated 53-μm (strut thickness) stent implanted in rabbit abdominal aorta; **b**, **c** mass loss and radial strength of nitride 70-μm stent implanted in rabbit abdominal aorta; **d** μCT images of coated 53-μm stent after 6 months of implantation in rabbit abdominal aorta; **e** μCT and histopathology of nitride 70-μm stent after 53 months of implantation in porcine coronary artery showing interstitial fluid (red arrow) between smooth muscle cells and somatic cell, and corrosion products migrating to adventitia. ▲  = strut footprint, △ = macrophage Adapted with permission from Elsevier (Lin et al. 2016, 2017)

Accelerating the in vivo corrosion of iron-based implants has been the focus of many works. The interestingly improved corrosion rates during in vitro corrosion tests are often not replicated in the in vivo tests. Drynda et al. (2015) observed that a series of Fe–0.5Mn, Fe–2.7Mn, and Fe–6.9Mn alloys exhibited no significant corrosion even after 9 months implanted subcutaneously in mouse. The formation of barrier (Fe–Mn phosphates) layers was determined as the cause of the high corrosion resistance, so strategies to dissolve these layers or to prevent their formation need to be developed to expedite the in vivo corrosion of Fe–Mn implants. Kraus et al. (2014) had similar finding when observing Fe–10 Mn–1Pd and Fe–21Mn–0.7C–1Pd pins implanted in a growing rat skeleton over a period of 1 year. They suspected that a dense layer of corrosion products acted as a barrier against oxygen transport, whereas oxygen is prerequisite for iron corrosion and its availability is rather limited in bony tissue. Thus, the corrosion of alloys should depend on hydrogen evolution, as it has a sufficiently low electrode potential, however, at a much lower rate than in presence of oxygen. More recent studies on long-term in vivo implantation of iron stents also indicated the need of increased corrosion rate. Lin et al. (2016) implanted 53 μm strut drug-eluting coronary stents made of nitride iron (+zinc barrier layer and 12 μm PLLA coating) in rabbit abdominal aorta. The stents maintained adequate scaffolding (125 kPa) after 3 months of implantation while having a shortened corrosion period to 13 months. However, a complete bioresorption of the corrosion products [Fe_3_O_4_, FeOOH, Fe_2_O_3_, and Fe_3_(PO_4_)_2_ was not observed (Fig. 3a, d)]. The same group reported another study that showed 70-μm strut nitride iron stents to lose its mass twice as much as pure iron stent after 36 months of implantation in rabbit abdominal aorta (Fig. 3b, c) (Lin et al. 2017). Although the study showed signs of good long-term biocompatibility (Fig. 3e), the complete corrosion of the stent and the absorbance of the insoluble corrosion products may take 4–6 years long, and thus, further increase of corrosion rate is necessary. The results obtained by Qi et al. (2018) showed that some PLLA-coated stents could totally corrode in the abdominal aorta of New Zealand white rabbits, whereas all the non-coated stents left some remnants of struts several months after implantation.

**Fig. 4:**
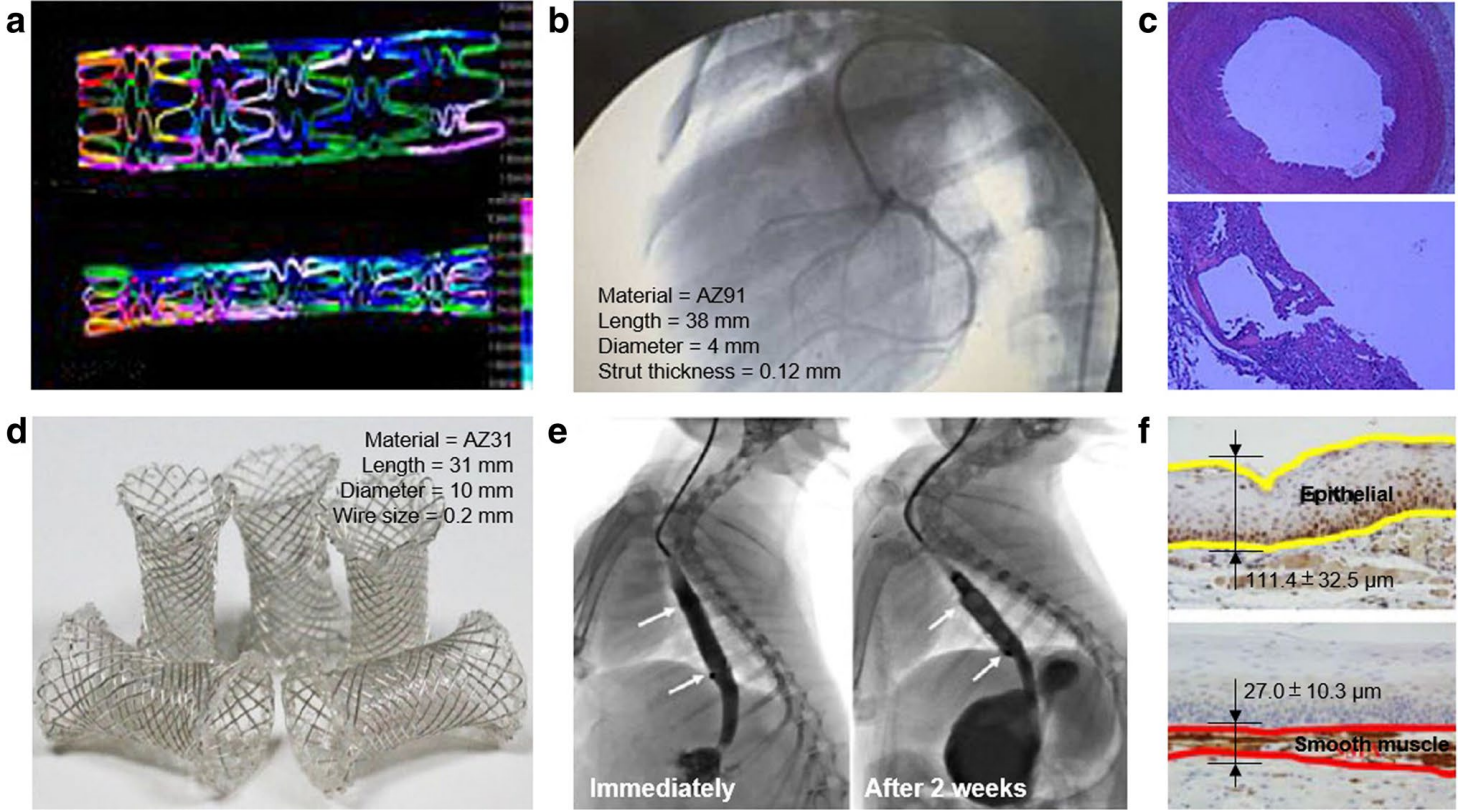
In vivo corrosion of magnesium stents: **a** simulation model of stent dilation and constriction, **b** X-ray angiography of stent implantation on coronary artery of dog, **c** histology of the stent in the arterial vessel after 28 days of implantation, **d** photographs of esophageal stents, **e** radiographs showing the stent patency is maintained 14 days after stent insertion in the normal esophagus of rabbits, and **f** esophageal wall remodelling with thin layer of epithelial (yellow line) and smooth muscle cell (red line) layers. Adapted with permission from John Wiley and Sage Publications (Yue et al. 2015; Zhu et al. 2016)

Although magnesium-based implants have demonstrated high potentials, fast corrosion is often observed in the in vivo studies. A recent study by Yue et al. (2015) acknowledged the need for longer period of corrosion of magnesium stents which can shed light on the incidence of late thrombosis and the extent of late lumen loss. Their 28-day-long implantation of Mg–Al–Zn alloy stent (Fig. 4a) in coronary artery of mongrel dogs (Fig. 4b) proved that the stent has good plasticity and strong resistance, and it induced mild neointimal hyperplasia 2–4 weeks after stenting (Fig. 4c). However, the short corrosion time of the stent could give some side effects such as the vascular elastic recoil and late lumen loss. Besides being targeted for endovascular stents, magnesium alloys were also utilized for esophageal stents. Zhu et al. (2016, 2017) successfully determined its feasibility through the implantation of silicone-covered magnesium esophageal stent (Fig. 4d) in the esophagus of rabbits. The stents showed good flexibility, elasticity, and patency (Fig. 4e), and they corroded slower than bare magnesium stents. The stents assisted the esophageal wall remodelling with desired thin epithelial and smooth muscle layers (Fig. 4f) and provided a reliable support for at least 2 weeks with acceptable migration rates without causing severe injury or tissue reaction when compared with plastic stents or collagen deposition.

**Fig. 5:**
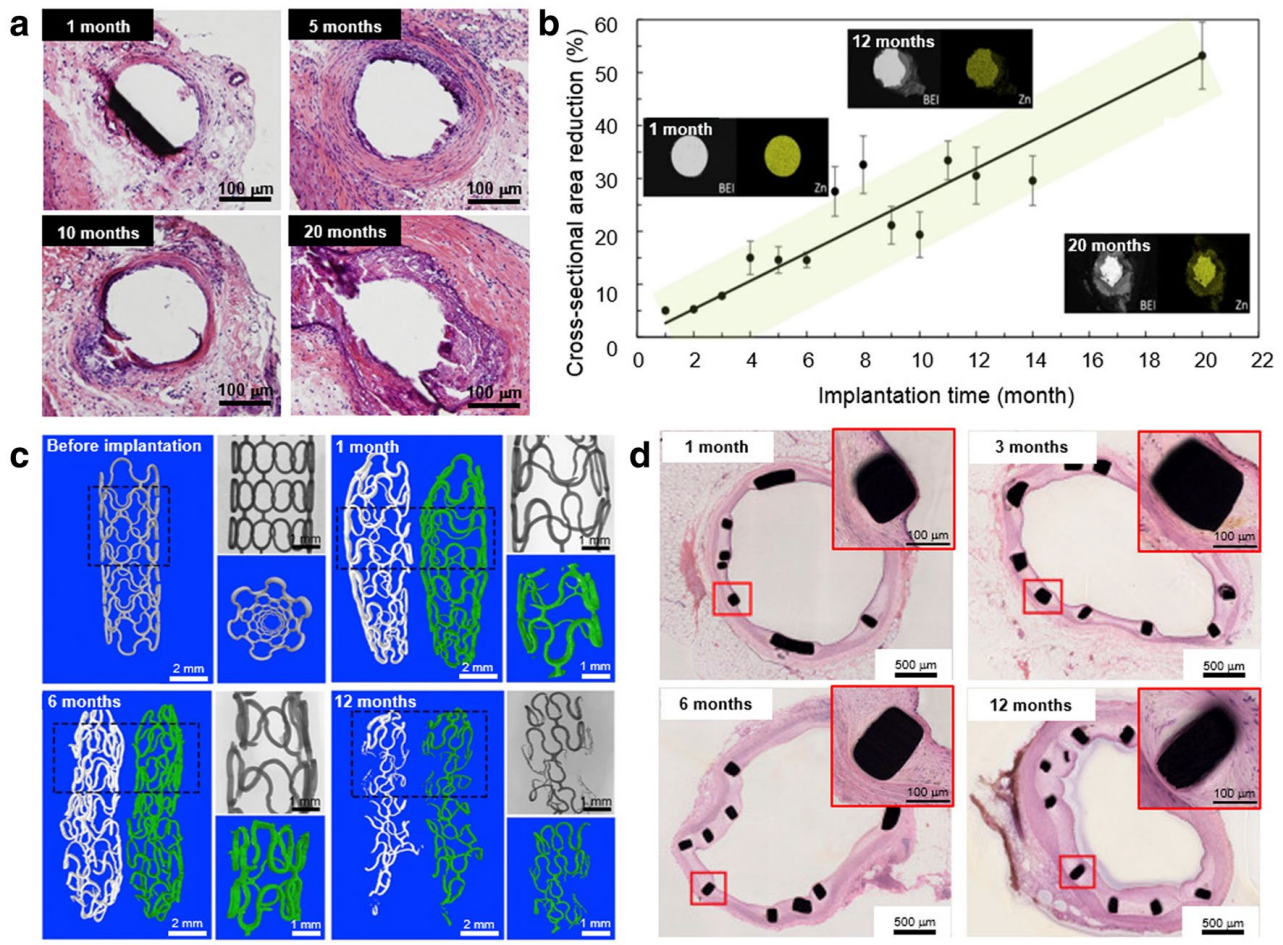
In vivo corrosion behavior of zinc: **a** histology of the abdominal aorta wall of murine after different time of implantation of the zinc wires, **b** cross-sectional reduction rate of the implanted zinc wires as a function of implantation time, **c** μCT images of zinc stents after different time of implantation in the abdominal aorta of Japanese rabbits with each showing: a 3D reconstruction among which the white one is the residue zinc stent and the green one represents corrosion products (left); 2D and 3D images of rectangular area of the stent (top right), and a magnified 3D image combining the residue zinc and corrosion products, and **d** histology of the aorta after different time of implantation of the zinc stents Adapted with permission from Elsevier (Drelich et al. 2017; Yang et al. 2017)

A different in vivo corrosion behavior was observed for zinc implants. In a long-term implantation study, Drelich et al. (2017) demonstrated that chronic inflammation in relation with corrosion activity of zinc wires implanted in the murine artery was subsided between 10 and 20 months (Fig. 5a). The wires exhibited a steady corrosion rate for up to 20-month postimplantation (Fig. 5b) without causing local toxicity despite a steady build-up of passivating corrosion products and intense fibrous encapsulation of the wire. This continued corrosion indicates that zinc stents could safely corrode within a time frame of approximately 1–2 years. Yang et al. (2017) reported the implantation of pure zinc stents in rabbit abdominal aorta and found that the stents conserved its mechanical integrity for 6 months and almost half of the stent volume was corroded after 12 months of implantation (Fig. 5c). They figured out that the corrosion process involved a conversion mechanism in a microenvironment that evolved from dynamic blood flow to neointimal tissue. The stent strut was covered by neointimal layer starting from the first month of implantation indicating a rapid endothelialization, but there was no intimal hyperplasia or obvious accumulation of corrosion products even after 12 months of implantation (Fig. 5d). These studies demonstrate an optimistic in vivo corrosion behavior of pure zinc.

### Correlation between in vitro and in vivo corrosion

A desirable corrosion behavior is mostly observed in the in vitro setting where a full control on the testing parameters can be established. Once the metals are exposed to the in vivo environment, they often behave differently (Sanchez et al. 2015; Bowen et al. 2015). In the in vivo environment, corrosion is influenced by the complex body fluid content (water, organic compounds, dissolved oxygen, anions, cations, amino acids, proteins, plasma, etc.) and the reciprocal interaction with the tissue response (Johnston et al. 2018). As the metal implantation causes injury, the body responses to it by decreasing the pH value around the implantation site (i.e., 5.3–5.6), and this in turn may accelerate corrosion process of the implant and reduce the local oxygen concentration (Paramitha et al. 2017). In their comprehensive review about factors influencing in vitro corrosion of magnesium and its pertinence to in vivo corrosion, Johnston et al. (2018) urged the inclusion of factors such as (i) proteins, (ii) amino acids, (iii) vitamins, and (iv) tissue encapsulation. At this point, there is a lack of knowledge on the best method to characterize the in vivo environment and corrosion mechanism and to correlate the results with the processing technique and properties of the metals. In addition, the correlation between the results of in vitro and in vivo corrosion experiments is still barely established (Zheng et al. 2014; Sanchez et al. 2015; Johnston et al. 2018).

After systematically reviewing the results of more than 20 in vitro and in vivo corrosion studies of magnesium and its alloys, Sanchez et al. (2015) found that deriving a correlation between in vitro and in vivo test results is yet challenging. This is attributed mainly to the difficulties in mimicking the complex in vivo physiological conditions with in vitro experiments and to the wide and variable testing parameters and procedures used in the studies. Myrissa et al. (2016) found that corrosion rates of pure magnesium, Mg–2Ag and Mg–10Gd alloys, are comparable between in vitro and in vivo conditions only after 4 weeks of experimentation period. In their work, the magnesium pins were immersed in DMEM (+ 10% FBS at 37 °C, 20% O_2_, 5% CO_2_, and 95% rH) and then measured for their mass loss, whilst similar pins were also implanted in Sprague–Dawley rats which then subjected to μCT scans for corrosion rate determination using the software Mimics^®^. Although the experiment indicated that the in vivo corrosion rate can be represented by in vitro rates to some extent, a more complex in vitro set-up (including biological component, mechanical and dynamical exposure of the metals) is needed to better mimic the in vivo condition.

The effect of dynamical exposure on corrosion behavior of absorbable metals was studied in two recent works. Wang et al. (2016b) analyzed the corrosion mechanism of Mg–Zn–Ca alloy under mimetic hydrodynamic conditions using an in situ and real-time electrochemical set-up in a vascular bioreactor. With this in vitro set-up, they demonstrated that flow-induced shear stress accelerated mass and electron transfer that led to an increase in uniform and localized corrosions. Effect of flow on corrosion of magnesium was also observed by Saad et al. (2016) using a test rig that mimicked the environment surrounding a cancellous bone where mass loss and mechanical integrity of the porous magnesium deteriorated linearly with an increase in porosity and degradation time. These studies confirm the influence of the in vivo condition, i.e., fluid flow, on corrosion behavior of absorbable metals. This is of a great importance to correlate the in vitro and in vivo corrosion behavior. The large variability between in vitro corrosion test methods makes comparison between studies increasingly difficult (Johnston et al. 2018). A standard approach is urgently needed to allow their searchers to observe the exact effect of a certain parameter of in vitro corrosion, and thus, a reliable iterative approach can be taken to improve the structure and processing of the materials.

### Real-time corrosion monitoring

The body responds to the corrosion of an absorbable metal implant via a cascade process of protein absorption, coagulation, acute inflammation, chronic inflammation, and foreign body response. Acute interaction occurs shortly after implantation. It induces the formation of an interspace between the implant and the tissue which then is filled with a large volume of body fluid. This is followed by cellular inflammation and then proliferation to finally fill the interspace (Kim et al. 2016). The body-response phenomena have been successfully evaluated using the tools adopted from those used for inert biomaterials. The common tools for assessing absorbable metal implants are radiography, ultrasonography, micro-computed tomography (μCT) and its advanced synchrotron version (SRμCT), magnetic resonance imaging (MRI), blood evaluation, and histological and implant retrieval analysis (Paramitha et al. 2017). However, these methods provide segmental, discontinued and offline results, so they are mostly unable to reveal the fundamentals of in vivo corrosion of the metals which is a continuous and time-dependent process. The nature of corrosion process of absorbable metals that produces metal and hydroxyl ions, hydrogen gas (for magnesium), and a flow of electric current should be exploited as the basis for developing in vivo corrosion assessment tools.

**Fig. 6:**
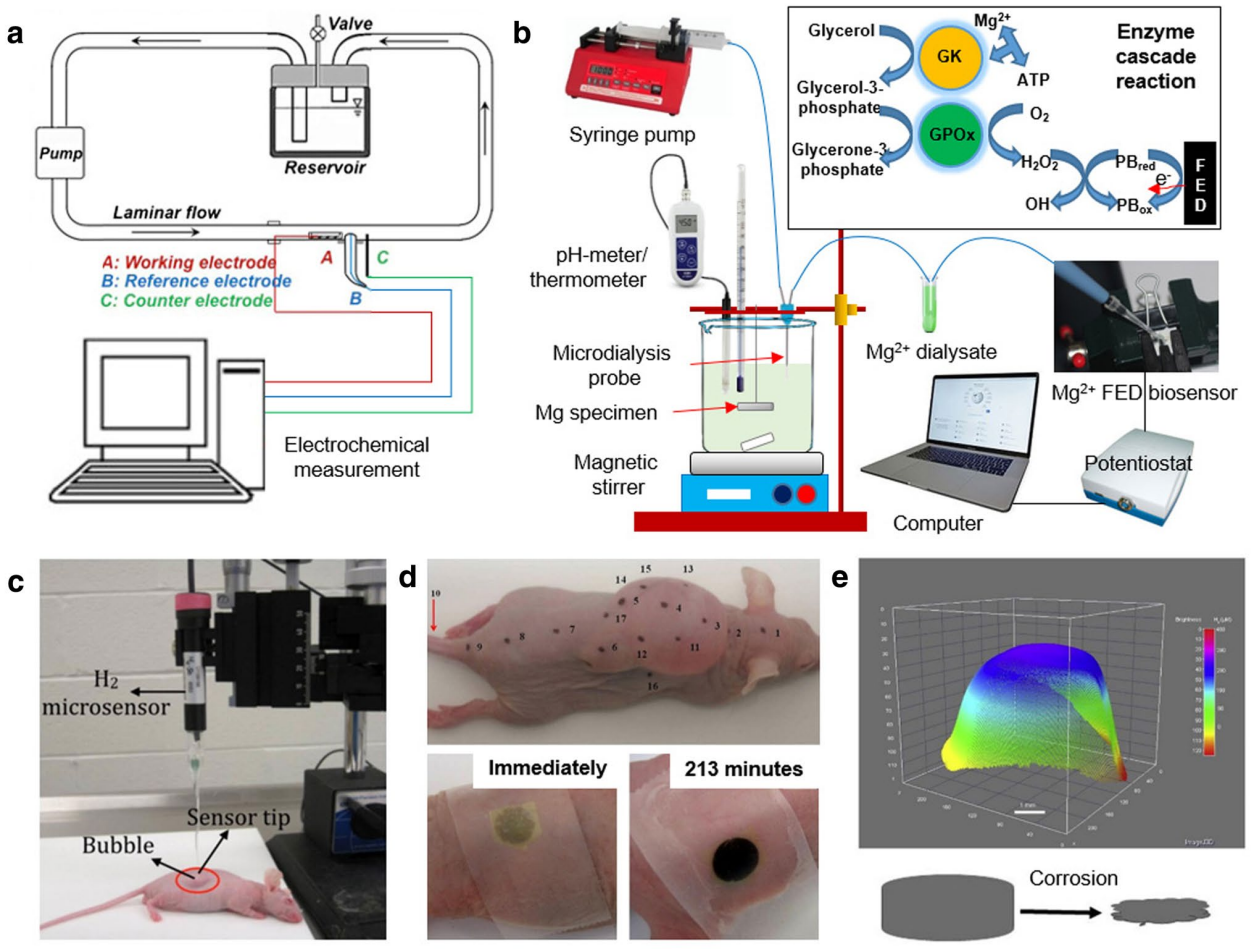
Corrosion monitoring systems: **a** experimental set-up of in situ and real-time flow-induced electrochemical corrosion study, **b** experimental set-up of the microdialysis probe-FED biosensor coupling with potentiostat (inset: dialyzate is dropped at the reaction zone of the FED that was immobilized with the GK and GPOx enzymes to detect Mg^2+^ ions via enzymes cascade reaction), **c** hydrogen microsensor assembled on a micromanipulator for measuring hydrogen transdermally from a magnesium alloy implanted subcutaneously in a mouse where the sensor tip is in a direct contact with the mouse skin, **d** photograph of an anaesthetized nude mouse with marked measurement points, and color development of thin-film visual hydrogen sensor at two different observation times, **e** 3D reconstruction of the brightness change in the hydrogen sensor area at 213 min and associated volume change of the magnesium implant. Adapted with permission from Elsevier and Springer Nature (Wang et al. 2016b; Zhao et al. 2016b, c; Natasha et al. 2018)

Over the past 5 years, several reports on the continuous assessment of corrosion behavior of absorbable metals have been published. Wang et al. (2016b) combined a vascular bioreactor that circulates simulated body fluid with an electrochemical cell that is connected to a potentiostat (Fig. 6a). With this set-up, they analyzed the in situ and real-time electrochemical corrosion mechanism of magnesium alloy under the influence of dynamic flow as previously described. Lately, Natasha et al. (2018) developed an online monitoring system which consisted of a microdialysis probe (as a tool to sample the fluid adjacent to magnesium implant) and a fabric-based electrochemical device (FED) (as a catalytic biosensor specific to Mg^2+^), both of which were connected to a potentiostat (Fig. 6b). The device demonstrated a pseudo-linear response of concentration vs. time from 0.005 to 0.1 mmol/L with a slope of 67.48 μA/mmol L. It also showed high ion selectivity with detectable interfering species less than 1%, a high temporal resolution, and a reduced sampling time (as it required only 3 μL of fluid sample to complete a measurement). This system could be further developed as a potential tool for real-time assessment of the in vivo corrosion behavior of magnesium implant.

Zhao et al. (2016a) developed a set-up composed of hydrogen gas sensor and capillary pH and Mg^2+^ microsensors to measure the real-time concentration of magnesium ion, hydroxyl ion, and hydrogen gas. By using the set-up, they were able to generate a map of hydrogen concentration in the vicinity of magnesium alloy sample. The set-up was further developed to a transdermally (non-invasive) electrochemical hydrogen microsensor (Fig. 6c) and tested for measuring in vivo corrosion of magnesium implants in mouse (Fig. 6d) (Zhao et al. 2016c). Using the sensor, they realized that hydrogen permeated through the skin at the concentration as low as 30–400 μM with a fast response time of 30 s. Although the hydrogen levels permeated through the skin were very low, the sensor changed its color to give a 3D visualization of hydrogen permeation (Fig. 6e) (Zhao et al. 2016b). When the transdermal hydrogen sensor measurement results were combined with those of ICP-MS and XPS, a more comprehensive understanding of in vivo corrosion behavior of magnesium was obtained. It was realized that the impurities were among the determinant factors responsible for rapid in vivo corrosion of magnesium (Zhao et al. 2017a). However, since it is a non-invasive sensor which depends on the hydrogen permeation through the skin, it may not be practical for monitoring in vivo corrosion of magnesium implants placed deeper beneath the skin, i.e., bone screw and plate implanted under the muscle.

The available in vitro corrosion data of absorbable metals could not be directly compared, as the test condition varies from one report to another. This points out the importance of using some standardized methods or common protocols that allow a comparison between results obtained in different labs. Many in vivo parameters are not yet mimicked in the in vitro corrosion testing, and thus, a correlation between corrosion rates obtained in vitro with those measured in vivo is difficult to establish. Thus, studies leading to a better understanding of in vivo corrosion behavior of absorbable metals should become a focus of the future works. In situ and real-time corrosion monitoring techniques based on the electrochemical nature of corrosion, e.g., using a minimally invasive sampling probe which allows a deeper sampling penetration adjacent to the implant, are worth further attentions. Aside from the major works on iron- and magnesium-based metals, there is a rapid increase of reports on zinc-based metals where pure zinc has been rapidly becoming an object of in vivo implantation studies, and some suggested its potential application for vascular stents. However, more basic research, i.e., on in vitro corrosion and cytocompatibility, is still needed to confirm the suitability of zinc-based metals especially its alloys for absorbable metals. The low mechanical properties of pure zinc were improved by alloying, yet the in vivo corrosion of zinc alloys is still unclear (Mostaed et al. 2016). For the iron-based metals, considering their very low corrosion rates and the phenomenon of barrier layer of corrosion product, its suitability for large temporary bone implants such as pins and screws appears questionable. Its usage for fine-structured implants such as stent or scaffold is, however, promising. As indicated in the two PLLA-coated stent studies (Lin et al. 2016; Qi et al. 2018) and the PLGA-infiltrated iron scaffold (Yusop et al. 2015), polymer coating with adjustable composition could become a way to regulate the in vivo corrosion of fine-structured iron-based implants. The in vivo studies on magnesium-based metals have acknowledged the limitation related to the fast corrosion of magnesium which only allowed an effective mechanical support for shorter period of time than is desired, so further research to delay the corrosion is mandatory. The following section describes the clinical studies of magnesium-based implants and the attempts to tailor their corrosion behavior.

## Translational research

Among all three classes of absorbable metals, only magnesium alloys have been subjected to the majority of basic research reported in the last decade and thus progressed to translational research. Published data for certain magnesium alloys, i.e., Mg–RE and Mg–Ca families, are available in full span from their metallurgy, and mechanical properties to their in vitro and in vivo corrosion. The translational research brings this knowledge to clinical use.

### Magnesium coronary stents

**Fig. 7:**
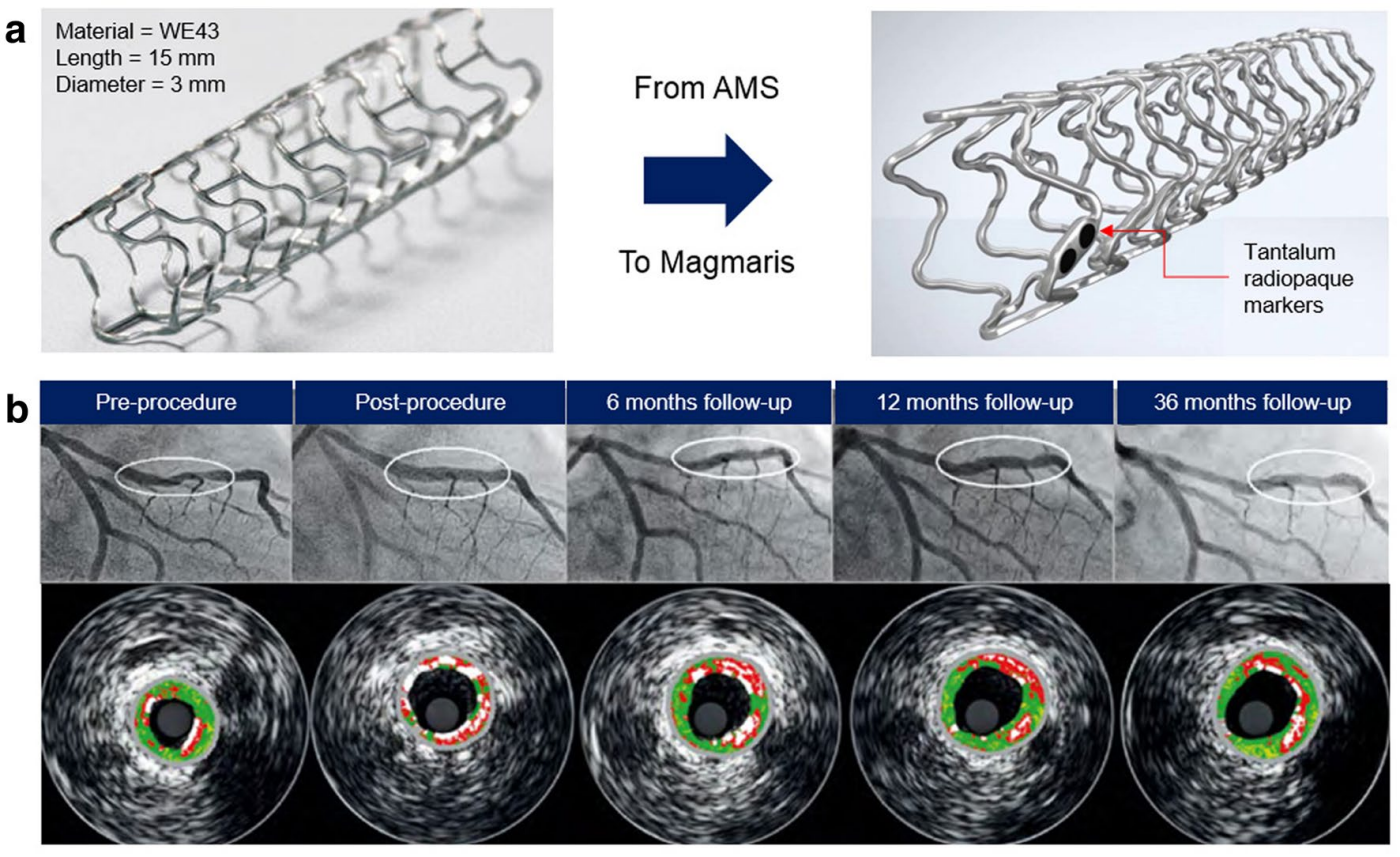
Clinical trials of magnesium coronary stents: **a** evolution from the first-generation absorbable magnesium stent to the CE Mark approved Magmaris stent; **b** serial quantitative angiographic and intravascular ultrasound of a patient with DREAMS 2G implantation before and after procedure and at different follow-up times demonstrating a good conformability to the vessel, detection of the scaffold corrosion at 6 months, homogeneous neointima formation at 12 months, and preservation of the lumen without any restenosis at 36 months. Adapted with permission from Europa Digital and Publishing (Haude et al. 2017)

Coronary stents made of Mg–RE alloy have been delivered by Biotronik (Berlin, Germany), and they have been scrutinized in a continuous set of clinical studies since 10 years ago. To slow down its corrosion, the stent was coated with PLGA containing paclitaxel drug. Therefore, it was named as the drug-eluting absorbable metal scaffold (DREAMS), and then, the stent was subjected to the first-in-man trial called BIOSOLVE-I at five European centres (Bartosch et al. 2015). The DREAMS was viewed as a good alternative to polymeric absorbable scaffolds, as, in 3-year follow-up, the overall long-term outcome was excellent, i.e., no cardiac death or scaffold thrombosis occurred (Haude et al. 2016a). The angiographic performance measures of DREAMS were still considered inferior to those of contemporary drug-eluting stents, and thus, the second generation device (DREAMS-2G) was developed (Fig. 7a). It was then assessed in the BIOSOLVE-II study to determine its safety and performance in symptomatic patients with de-novo coronary artery lesions. In 6-month follow-up, the DREAMS-2G showed a substantially improved performance with a superior safety profile to that of its DREAMS-1G precursor, i.e., no thrombosis or cardiac death up to 6 months (Haude et al. 2016b). This desirable profile was continuously observed up to 12 months with stable angiographic parameters (Haude et al. 2016c). Additional evidence on the excellent safety profile of DREAMS-2G was provided by the 24 months of clinical outcomes after implantation where the definite or probable scaffold thrombosis was absence (Fig. 7b) (Haude et al. 2017). With these encouraging clinical results, the company landed CE Mark approval in 2016 for their absorbable magnesium stent named Magmaris. The stents will be soon available in the wider market, and it will offer the benefits of temporary arterial scaffolding for the patients.

### Magnesium bone screws

**Fig. 8:**
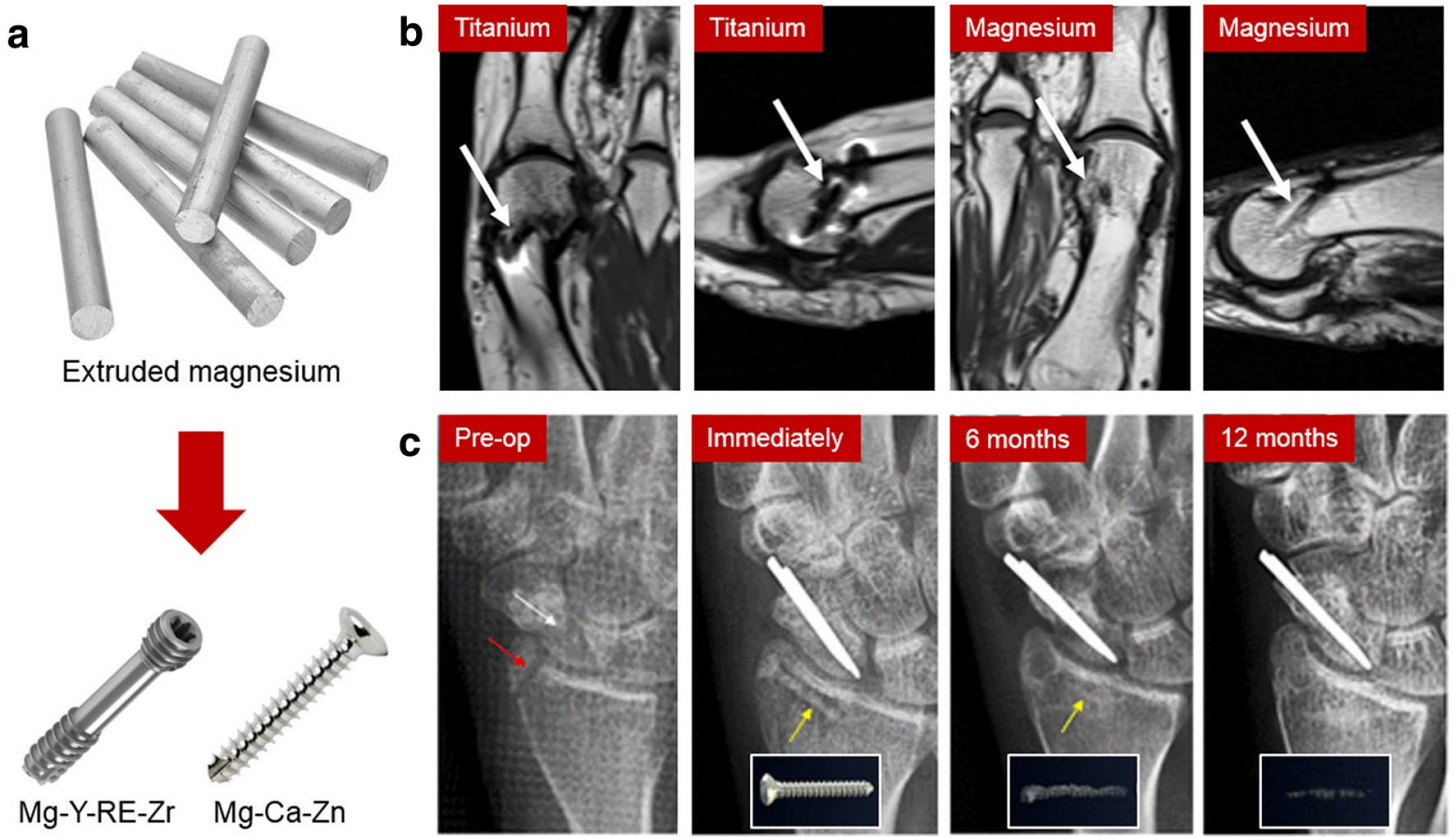
Clinical trials of magnesium bone screws: **a** transformation of extruded rod into bone screws; **b** MRI of forefoot 3 years after a modified chevron osteotomy showing the complete healing of osteotomies where more artifacts are observed on the titanium screw than the remnants of the magnesium screw. The white arrow indicates a hypointense structure found in the former position of the magnesium screw; **c** radiographs of the distal radius fracture (red arrow) and the scaphoid non-union (white arrow) of a 29-year-old female patient before and after the surgical procedures with the screw (yellow arrow) and the scaphoid non-union with stainless steel implant, showing corrosion progress and bone healing. Insets show the change of the screw over time. Adapted with permission from National Academy of Sciences and Elsevier (Lee et al. 2016; Plaass et al. 2018)

Magnesium bone implants, such as bone screws, achieved a relatively rapid clinical translation and commercialization (Fig. 8a). Many in vitro and in vivo studies demonstrated the promising applicability of magnesium alloys for bone implants. The clinical studies were conducted and reported by at least two groups in Germany and Korea and a few groups in China (Zhao et al. 2017b). In Germany, Plaass et al. (2018) reported a clinical study, wherein the Mg–Y–RE–Zr screw was compared with the standard titanium screw for fixation of a modified distal metatarsal osteotomy in 26 patients with a symptomatic hallux valgus (Fig. 8b). The 3-year postoperative MRI demonstrated a significant improvement for all tested clinical scores, i.e., AOFAS, SF-36, FAAM, and Pain-NRS, from preoperative to postoperative investigation. As no statistically relevant difference was found between the groups, the clinical results of the magnesium screw are comparable to those of the standard titanium screw. Less artifacts in the MRI was shown for magnesium implant without any implant related cysts, and the implant was corroding 3 years postoperatively. In a larger prospective clinical study involving 44 patients, they showed the feasibility of using the screws in hallux valgus surgery and advised surgeons to be aware of the proper handling of these implants and to know about corrosion effects during healing and its radiographic appearance (Plaass et al. 2016). These magnesium screws are now commercialized by Syntellix (Hanover, Germany) under the name Magnezix after receiving the CE Mark approval in 2017 (Biber et al. 2017).

Lee et al. (2016) reported the results of a long-term clinical study of Mg–5Ca–1Zn alloy screws in 53 distal radius fracture fixation cases performed at Ajou University Hospital (Suwon, Korea). Two conventional stainless steel pins were also used to fix a scaphoid fracture. Cortical continuity was observed after 6 months, while the diameter of the screw was significantly reduced (Fig. 8c). After 12 months of implantation, the distal radius fracture was completely healed, while the remaining screw was nearly impossible to differentiate from new surrounding bones. The patients did not feel any discomfort or pain throughout the entire study. They returned to their everyday life without any sign of pain or decrease in range of motion, and they gained back a normal range of grip power. The controlled corrosion of the alloy results in the formation of biomimicking calcification matrix at the corroding interface and initiation of the bone formation process. This facilitates the early bone healing process and leads to the complete replacement of the implant by the new bone tissue. After being approved by the Korean FDA in 2015, the Mg–5Ca–1Zn alloy screws are commercialized by U&i Corp. (Gyeonggi-do, Korea), and they are now available in the market as K-MET bioresorbable bone screws.

As commercial products, the exact materials’ composition and fabrication process of the aforementioned absorbable magnesium implants are not publicly available in scientific publications. However, one may have a look at patent databases such as that of the United States Patent and Trademark Office (USPTO). The following examples are three US patents assigned to the three companies who already commercialized the absorbable metals technology. First, the US Patent #US9700652B2 was assigned to Biotronik claiming an absorbable medical implant made of fiber-reinforced magnesium or fiber-reinforced magnesium alloys (Loeffler et al. 2017). It is comprised of a matrix of crystalline magnesium or magnesium alloy reinforced by amorphous or nanocrystalline fibers made of Mg-(10–40 wt%) Zn-(0–20 wt%)*X*, wherein *X* is selected from the group of lanthanum, yttrium, silicon, aluminum, and calcium. This composite-like structure allows an increased strength and tunable corrosion behavior which are desirable for coronary stent applications. Second, Syntellix assigned the US Patent # US9402669B2 which claims a method for producing a medical implant, such as bone screw, nail, pin, plate, and suture anchor, from a magnesium alloy which contains 2.5–5 wt% RE, 1.5–5 wt% yttrium, 0.1–2.5 wt% zirconium, and 0.01–0.8 wt% zinc (Neubert and Schavan 2016). The method consists of melting the alloy at 700–900 °C, atomizing the molten alloy into alloy powder under a protective-gas atmosphere, shaping the powder into a green body, extruding it at 300–400 °C to obtain a molded part, and finally producing the medical implant from it. Third, the US Patent US20170119922A1 was assigned to U&I claiming a magnesium alloy for forming a medical implant, comprising 0–23 wt % calcium and 0–10 wt % zinc (Koo et al. 2017). The alloy possesses controlled corrosion resistance properties due to the electrical potential difference between magnesium phase and Mg_2_Ca phase which is not greater than 200 mV as measured in a biomimetic solution specified in the patent. As can be seen, the inventions claimed in those patents based on the material composition that leads to a controlled corrosion resistance, the composite structure that leads to an improved strength, and the processing method that results into a molded semi-finished material.

## Development of standards

As is required for all medical implants, an absorbable metal implant must demonstrate a predictable level of safety. Therefore, aside from meeting the basic needs of the intended implant application, additional absorbable-related standards can be useful for obtaining guidance regarding the appropriate evaluation of an implant’s absorbable-specific aspects. All standards are intended to facilitate the spread of the relevant knowledge, to assure the dissemination of innovative advances in technology, to smooth the trade between nations, and to share both good management and conformity assessment practices.

While multiple standards relevant to absorbable materials are under development, currently, there exist three published absorbable-implant-related standards that contain aspects relevant to absorbable metals: the ISO/TR 37137:2014: *Cardiovascular biological evaluation of medical devices* - *Guidance for absorbable implants* (ISO 2014a), the ISO/TS 17137:2014: *Cardiovascular implants and extracorporeal systems—*(ISO 2014b), and the ASTM F3036-13: *Standard guide for testing absorbable stents* (ASTM 2013). Although these somewhat general absorbable standards can be useful when evaluating absorbable metal implants, more comprehensive absorbable metal-specific guidance is needed. Realizing this need, both ASTM and ISO have been undertaking a collaborative effort to develop coordinated standardized guidance to appropriately evaluate the metallurgy, corrosion, and biocompatibility of absorbable metals (Hayes 2016). A draft of the coordinating “umbrella” document ISO/DTS 20721 will then link the four other absorbable metal-specific documents as described in Table 3.

**Table 3:** Specific standards under development for absorbable metals

Document type	Title of standard	Status
Umbrella document	ISO/DTS 20721: Implants for surgery—guidance for the assessment of absorbable metal implants(as revised)	Under development in ISO/TC 150
Materials document	ASTM F3160-16: Standard guide for metallurgical characterization of absorbable metallic materials for medical implants	Published 2016
Degradation document	ASTM WK52640: Standard guide to in vitro degradation testing of absorbable metals	Ballot approved; expected publication 2018
Fatigue document	ASTM WK61103: Standard guide for corrosion fatigue evaluation of absorbable metals	Updated draft review May 2018
Biological evaluation document	ISO/NP TS 37137-1: Biological evaluation of medical devices—Part 1: Guidance for absorbable implants	Under development in ISO/TC 194
	ISO/DTR 37137-2: Biological evaluation of medical devices—Part 2: Guidance for absorbable metal implants	Under development in ISO/TC 194

The ASTM F3160-16 is a published standard developed by the Medical and Surgical Devices Subcommittee F04.12 (Metallurgical Materials) (ASTM 2016a). It provides guidance to appropriately characterize and evaluate the chemical, physical, microstructural, and mechanical properties of absorbable metals, including inspection guidelines for wrought and cast metals. Subcommittee F04.15 (Materials Testing) is currently developing Work Item WK52640 (ASTM 2016b), which is intended to outline appropriate experimental approaches to conduct an in vitro degradation test on absorbable metal samples or devices. It provides guidance for appropriately controlling the test environment and includes optional use of a standardized degradation control material to facilitate comparison and normalization of results across laboratories. A third ASTM standard under development is Work Item WK61103 about corrosion fatigue evaluation which is now entering a very active development stage (ASTM 2018). None of the aforementioned ASTM standards include any in vivo or biocompatibility evaluation related guidance. While TC 150 (Implants for surgery) is sponsoring development of the overall guide for assessing absorbable metal implants, TC 194 (Biological and clinical evaluation of medical devices) is currently revising ISO/TR 37137:2014. The aim is to provide a general guidance for all absorbable implants, with an accompanying re-designation as ISO/TS 37137-1 (ISO 2018b). The committee is also actively drafting a text for the new ISO/TR 37137-2 (ISO 2018a).

The standards which are under development by ISO and ASTM will serve as a platform to help addressing the need for standardized in vitro corrosion test methods that allow the comparison of results from different labs. Once all these standards become active, they will certainly facilitate a rapid translation of absorbable metals technology toward both their clinical use and commercialization. This will be accompanied by economic benefits that can be realized when tangible improvement is made to patient’s quality of life.

## Conclusion and perspective

Today, magnesium alloys are considered as the most developing absorbable metals with a large data set obtained from both basic and translational research. The high in vivo corrosion rate of these alloys has been decreased by proper alloying and advanced metallurgical processes. Magnesium alloys are now used for producing the commercial coronary stents and bone screws and pins. Iron-based metals have been studied in many basic studies, but the results indicate that their usage in solid/bulky form has appeared questionable due to their low in vivo corrosion rate. Results from basic research on zinc-based metals indicate in vivo corrosion rates that fall between those of magnesium and iron. Pure zinc was quickly subjected to several in vivo studies which provided results that indicated its potential for endovascular stent material. Among the top remaining challenges in absorbable metals research is correlating the in vivo corrosion of absorbable metals with the in vitro corrosion and with the structure and processing of materials. The development of specific standardized guidance for absorbable metals is progressing, and it will help the researchers to address the remaining challenges while fostering a rapid clinical translation for the benefit of patients.

Finally, it seems more appropriate to use the term “absorbable” instead of “biodegradable” as it has been used in the ASTM and ISO standards and is acceptable by a larger material science community. The development of new tools capable of real-time depiction of the underlying phenomena of in vivo corrosion should be considered as one of the main directions for future works. More translational research should be encouraged for magnesium-based metals to cultivate the well-developed basic research data available in the literature. The over-claim on the clinical suitability of zinc-based metals seems to be premature as more confirming evidence from basic research is still needed, while, for iron-based metals, they seem to provide an optimum compromise of mechanical properties and corrosion behavior if used in their fine 3D structure such as stent and porous form.
